# Workplace Relations and Opportunities for Career Development Impact the Retention of Veterinarians in Shelter Medicine

**DOI:** 10.3389/fvets.2021.732105

**Published:** 2021-08-25

**Authors:** Lauren Powell, Chelsea L. Reinhard, James Serpell, Brittany Watson

**Affiliations:** School of Veterinary Medicine, University of Pennsylvania, Philadelphia, PA, United States

**Keywords:** shelter medicine, retention, veterinarian, job satisfaction, professional fulfillment

## Abstract

Shelter medicine has grown considerably over recent years with many shelters hiring veterinarians for the first time or expanding their veterinary teams. As a result, there is a dearth of shelter veterinarians and retention has become a key concern for the field. The goal of this study was to describe veterinarians' perceptions of shelter medicine, and their feelings of job satisfaction, loneliness, and professional fulfillment. The sample included 52 shelter veterinarians, 39 previous shelter veterinarians and 130 non-shelter veterinarians (*n* = 221) who each completed an online survey. Current and previous shelter veterinarians had comparable perceptions regarding the appeal of most shelter medicine duties, although there were differences in the duties they performed within their job. More current shelter veterinarians participated in population management, policy development, administrative duties, and decision-making for individual patients (euthanasia, treatment, and adoptability). Considering other employment attributes, we found previous shelter veterinarians had lower mean rankings than current and non-shelter veterinarians regarding their interactions with administrative staff, ability to be part of a multiple veterinarian team and the availability of mentorship. Loneliness and professional fulfillment were mostly comparable between the groups, although previous shelter veterinarians were more likely to report they felt unhappy (*X*^2^ = 16.60, *p* = 0.02) and left out at work (*X*^2^ = 12.43, *p* = 0.02). Our findings suggest veterinarians who participate in decision-making for patients and shelter management procedures may be more willing to continue working in shelter medicine. Animal shelters should also employ strategies to improve workplace relationships and offer career development opportunities to improve job satisfaction and retention of veterinarians within the field.

## Introduction

Shelter medicine was formally recognized as a specialty of veterinary medicine by the American Board of Veterinary Practitioners (ABVP) in 2014 (1). Shelter practice differs from traditional companion animal medicine as shelter veterinarians support the health and welfare of individual shelter animals, the population of shelter animals, animals within the community, and public health (2). The responsibilities of shelter veterinarians are wide ranging, including individual patient care, behavior evaluation, population management, disaster response, policy development for preventative health care, cruelty investigations, and community education (2, 3).

The field of shelter medicine has grown steadily over recent years as shelters increasingly recognize the need for veterinarians to maintain wellness and prevent disease in the shelter environment (2). Many shelters are hiring veterinarians for the first time or expanding their veterinary team (3, 4). Demand is also growing for veterinarians in low-cost spay/neuter clinics and access-to-care community clinics (4). As a result, there is currently a shortage of veterinarians in shelter medicine and retention of shelter veterinarians has become a crucial concern for the field (3, 4).

Animal shelters have implemented a number of strategies to increase recruitment and retention in shelter medicine (4). The average salary of shelter veterinarians has increased considerably over recent years (3) and is now equivalent to the median salary of private practice veterinarians (5). The field has also seen an increase in the provision of benefits, such as health insurance and paid continuing education leave (3). However, preliminary evidence shows shelter veterinarians continue to leave the field due to poor relationships with management, poor work/life balance, internal criticism, inadequate staffing/budget, and inadequate input in operations (4). Occupational stress, burnout, and compassion fatigue (a unique form of stress and burnout in which individuals have a reduced capacity to show empathy) are also key concerns for the field of veterinary medicine (6). Shelter veterinarians may be particularly susceptible to feelings of stress and burnout due to euthanasia-related duties within their role and the caring-killing paradox, i.e., the notion that shelter staff must kill the animals for whom they have been providing care (7). On the other hand, high levels of job satisfaction and professional fulfillment, which includes feelings of happiness, engagement, and meaningfulness at work (8), can reduce feelings of burnout in the veterinary profession (9).

There is negligible existing research that has investigated characteristics of employment in shelter medicine relative to turnover or retention of shelter veterinarians. Understanding these characteristics is crucial to the continued growth of the field, so the aim of this study was to investigate veterinarians' perceptions of common duties and attributes of employment in shelter medicine, and veterinarians' feelings of job satisfaction, loneliness, and professional fulfillment.

## Methods

### Protocol

Veterinarians were recruited to participate in this study between September 1st 2020 and March 1st 2021 through social media postings, relevant industry groups, and email listservs, such as the Association of Shelter Veterinarians' listserv and the American Association of Veterinary Medical College's (AAVMC) Primary Care Veterinary Educators listserv. The study was also shared in the University of Pennsylvania School of Veterinary Medicine alumni newsletter and the ABVP newsletter. Veterinarians from all fields of veterinary medicine were eligible to participate in the study and were categorized into 3 groups based on their employment history: (1) current shelter veterinarians who were employed in shelter medicine at the time of completing the survey; (2) previous shelter veterinarians who worked in shelter medicine previously but were not working in the field at the time of completing the survey; and (3) non-shelter veterinarians who were qualified veterinarians who had never worked in shelter medicine. We included current and previous shelter veterinarians to identify characteristics of shelter medicine that may be related to retention in the field. The non-shelter veterinarians served as a control group. The study was exempt from review by the University of Pennsylvania Institutional Review Board (Protocol No. 843889). All study participants provided informed written consent prior to completing the survey.

### Questionnaire

Qualtrics was used to administer the questionnaire, and all responses were recorded anonymously. The full questionnaire is provided in the Supplementary Material and included questions under 3 main sections: (1) demographics and employment characteristics; (2) perceptions of shelter medicine duties and attributes of employment in shelter medicine; and (3) job satisfaction, loneliness and professional fulfillment. The demographic questions included the participant's age, gender, race, ethnicity, education, and student loan debt. Participants were then asked questions about their current employment including the field of veterinary medicine, employment type (full time, part-time, etc.), length of employment, and salary.

The second part of the questionnaire required participants to rate the appeal of 25 common duties of shelter medicine on a 5-point scale from very unappealing (1) to very appealing (5). We then asked participants to rate the influence of 23 characteristics of shelter medicine on their willingness to work in the field, ranging from strongly discourage (1) to strongly encourage (5). These questions were developed based on the core duties and tasks of shelter veterinarians identified through the 2007 DACUM (Developing A Curriculum) analysis and described in the ABVP applicant handbook (2), as well as previous research from Kreisler, Spindel et al. (3), and the experiences of the authors and other experts within the field. Each of the duties and characteristics of shelter medicine were presented in a randomized order in Qualtrics to avoid possible order effects.

The final section of the questionnaire asked participants to rate their overall job satisfaction on a 5-point scale from very dissatisfied (1) to very satisfied (5). Participants were also asked if they would change the number of hours they worked per week. Possible answers included ‘work fewer hours for less compensation’, ‘work more hours for more compensation’ or ‘work the same number of hours for the same compensation’(10). We also included 3 questions about feelings of loneliness at work from the UCLA-3 loneliness scale (a valid and reliable tool to assess loneliness (11)), whereby respondents had to indicate how often they felt each statement was applicable to them. Answers ranged from hardly ever (1), to some of the time (2), and often (3). A UCLA-3 score was calculated as the sum of all items (11). Finally, we included 6 statements from the professional fulfillment scale of the Professional Fulfillment Index (PFI), such as “I feel in control when dealing with difficult problems at work” (8). Participants could respond to each statement on a 5-point scale from not at all true (0) to completely true (4). A professional fulfillment scale score was then calculated as the average of the 6 items. Previous shelter veterinarians were instructed to answer these questions regarding job satisfaction, loneliness, and professional fulfillment in reference to their previous role.

### Statistical Analysis

Statistical analyses were conducted in SPSS (IBM SPSS Statistics version 27). Pearson Chi-Square tests, or Fisher Exact tests where more than 20% of cells had expected values <5, were used to examine differences in demographic characteristics between the groups. Kruskal-Wallis tests with *post hoc* analyses including Bonferroni correction were used to compare the median responses of shelter veterinarians, previous shelter veterinarians, and non-shelter veterinarians regarding the appeal of common shelter medicine duties and the importance of employment attributes. Kruskal-Wallis tests were also used to investigate job satisfaction and UCLA-3 loneliness scores between the groups of veterinarians. A one-way ANOVA was used to compare the professional fulfillment scale score between current, previous, and non-shelter veterinarians. Responses to individual items within the UCLA-3 loneliness scale and the professional fulfillment scale were assessed using Pearson's Chi Square. Pearson's Chi Square/Fisher Exact tests were also used to examine the relationship between veterinarians' student loan debt and the importance of salary, employee benefits and loan forgiveness programs in shelter medicine, as well as the relationship between desired work hours and the importance of the regularity of work hours, number of work hours, the appeal of being on-call and the appeal of working on weekends. *P* < 0.05 was considered statistically significant.

## Results

### Demographic Characteristics

Fifty-two shelter veterinarians, 39 previous shelter veterinarians and 130 non-shelter veterinarians completed the survey, including graduates from 46 veterinary medical university programs across Australia, Canada, United States, Scotland, England, West Indies, New Zealand, Italy, the Netherlands, and the Philippines. The University of Pennsylvania (36%) and Cornell University (9%) were the most represented universities. The non-shelter veterinarians in this study primarily worked in small animal practice (*n* = 74, 57%) and academia (*n* = 36, 28%), although there were a few veterinarians from mixed practice (*n* = 3), equine (*n* = 4), exotics (*n* = 2), laboratory animal (*n* = 4), research (*n* = 3), and regulatory/policy (*n* = 2). One large animal veterinarian and 1 government veterinarian also completed the survey. Of the 39 previous shelter veterinarians, 25 had moved to small animal practice, 12 had moved to academia, 1 worked in government and 1 worked in regulatory/policy. Most previous shelter veterinarians had left the field 3–5 years ago (*n* = 13, 33%) or <1 year ago (*n* = 10, 26%), although some veterinarians had left shelter medicine more than 20 years prior.

The descriptive characteristics of the sample are provided in Table 1. There were no significant differences in gender (*X*^2^ = 5.76, *p* = 0.16), age (*X*^2^ = 11.60, *p* = 0.07), or race (*X*^2^ = 13.77, *p* = 0.17), although salary differed between current, previous, and non-shelter veterinarians (*X*^2^ = 27.61, *p* = 0.001). *Post hoc* analysis with standardized residuals showed current shelter veterinarians were more likely to earn $50,000–$99,999, while non-shelter veterinarians were more likely to earn <$50,000 or $100,000–$149,999. There were no significant differences in outstanding student loan debt (*X*^2^ = 6.53, *p* = 0.32) or student loan debt at the time of graduation between the groups (*X*^2^ = 2.13, *p* = 0.94).

**Table 1:** Descriptive characteristics of study sample.

	**Shelter veterinarians (** ***n*** **=52)**		**Previous shelter veterinarians (** ***n*** **= 39)**		**Non-shelter veterinarians (** ***n*** **= 130)**	
**Characteristics**	**%**	***N***	**%**	***N***	**%**	***N***
**Gender**						
Female	90.4	47	74.4	29	86.2	112
Male	9.6	5	25.6	10	13.1	17
Other	0	0	0	0	0.8	1
**Age**						
20–29 years	19.2	10	5.1	2	21.5	28
30–39 years	36.5	19	28.2	11	32.3	42
40–49 years	26.9	14	23.1	9	20.0	26
>49 years	17.3	9	43.6	17	26.2	34
**Race**						
American Indian/Alaskan Native	0	0	0	0	1.5	2
Asian	1.9	1	5.1	2	0	0
Black/African American	0	0	0	0	0.8	1
Hispanic/Latino	1.9	1	2.6	1	4.6	6
White	94.2	49	82.1	32	90.0	117
Mixed race	1.9	1	2.6	1	1.5	2
Prefer not to answer	0	0	7.7	3	1.5	2
**Current student loan debt**						
<$50,000	46.2	24	66.7	26	57.7	75
$50,000–$149,999	21.2	11	12.8	5	20.0	26
≥$150,000	32.7	17	17.9	7	20.8	27
Prefer not to answer	0.0	0	2.6	1	1.5	2
**Student loan debt at graduation**						
<$50,000	30.8	16	33.3	13	36.9	48
$50,000–$149,999	28.8	15	33.3	13	26.9	35
≥$150,000	40.4	21	33.3	13	34.6	45
Prefer not to answer	0.0	0	0.0	0	1.5	2
**Salary**						
<$50,000	3.8	2	10.3	4	**15.4**	**20**
$50,000–$99,999	**55.8**	**29**	35.9	14	20.8	27
$100,000–$149,999	36.5	19	38.5	15	**39.2**	**51**
≥150,000	1.9	1	10.3	4	16.2	21
Prefer not to answer	1.9	1	5.1	2	8.5	11

*Bold text indicates there was a statistically significant difference based on Pearson Chi-Square (p < 0.05)*.

For both current and previous shelter veterinarians, the length of employment in shelter medicine ranged from <1 year to more than 20 years. For current shelter veterinarians, 27% had been employed for 3–5 years, 23% had been employed for 5–10 years and 25% had been employed for 11–20 years. Among previous shelter veterinarians, 31% were employed for 1–2 years, 28% were employed for 3–5 years and 21% were employed for 11–20 years prior to leaving the field. There were no significant differences in the length of employment between previous and current shelter veterinarians (*X*^2^ = 6.66, *p* = 0.25).

There were no significant differences between current, previous, and non-shelter veterinarians (*X*^2^ = 6.75, *p* = 0.16) when asked if they would change the number of hours they worked per week. Most veterinarians would choose to continue working the same number of hours with no change to their compensation (59%), although 25 % would prefer to work fewer hours for a lower level of compensation and 16% would prefer to work more hours for a higher level of compensation.

### Likelihood of Future Employment in Shelter Medicine

Most non-shelter veterinarians were extremely unlikely (41%) or somewhat unlikely (22%) to consider working in shelter medicine in the future, while 14% were somewhat likely and 11% were extremely likely to consider future employment in the field. Comparatively, 21% of previous shelter veterinarians were extremely unlikely and 18% were somewhat unlikely to work in shelter medicine in the future. A larger portion of previous shelter veterinarians were somewhat likely (23%) or extremely likely (28%) to consider working in shelter medicine.

### Duties of Shelter Medicine

Table 2 displays the median appeal of common shelter medicine duties for current, previous, and non-shelter veterinarians. There were no significant differences between current and previous shelter veterinarians in the appeal of most duties, with one exception: population management. Previous shelter veterinarians reported a significantly lower mean rank regarding the appeal of population management compared with current shelter veterinarians. Non-shelter veterinarians reported significantly lower mean ranks than current and previous shelter veterinarians across a number of duties, including spay/neuter, pediatric spay/neuter, other surgery, and the development of health care policies or standard operating procedures (SOP). Non-shelter veterinarians also rated population management, humane euthanasia, euthanasia decision-making, administrative responsibilities, being on-call for emergencies, forensics/cruelty investigations, and testifying in court as significantly less appealing compared with current shelter veterinarians.

**Table 2:** Kruskal-Wallis tests describing differences in appeal of duties of shelter medicine between current, previous and non-shelter veterinarians.

	**Current shelter veterinarians**		**Previous shelter veterinarians**		**Non-shelter veterinarians**		***X*^**2**^**	***P* value**
	**Median (IQR)**	**Mean rank**	**Median (IQR)**	**Mean rank**	**Median (IQR)**	**Mean rank**		
Spay/neuter	5 (4–5)	142.29	5 (4–5)	134.64	**4 (2–5)**	**91.39**	**32.93**	**<0.001**
Pediatric spay/neuter	5 (4–5)	150.13	4 (3–5)	131.50	**3 (2–4)**	**89.20**	**40.89**	**<0.001**
Other surgery	5 (4–5)	134.67	4 (3–5)	125.99	**4 (2–4)**	**97.03**	**16.65**	**<0.001**
Population management	4 (3–5)	144.37	**4 (3–5)**	**112.32**	**3 (2–4)**	**97.26**	**21.36**	**<0.001**
Humane euthanasia	3 (3–3)	131.11	3 (2–3)	108.63	**2 (1–3)**	**103.27**	**8.57**	**0.01**
Euthanasia decisions	3 (3–3)	130.24	3 (2–3)	110.81	**2 (1–3)**	**103.36**	**7.24**	**0.03**
Administrative responsibilities	3 (2–4)	130.24	3 (1–4)	113.32	**2 (1.75–3)**	**102.61**	**7.46**	**0.02**
Physical exams	4 (4–5)	119.03	4 (3–5)	118.27	4 (3–4.25)	105.61	2.54	0.28
Treatment decisions	4.5 (4–5)	124.42	4 (4–5)	121.41	4 (4–5)	102.51	**6.56**	**0.04**
Adopt–ability decisions	4 (3–4)	124.63	3 (3–4)	117.05	3 (2–4)	103.73	4.71	0.10
Behavior evaluations	4 (3–4)	126.97	3 (3–4)	110.99	3 (2–4)	104.62	4.87	0.09
Developing health care policies and/or SOPs	4 (3.25–5)	134.89	4 (3–5)	127.15	**3.5 (2–4)**	**96.60**	**17.52**	**<0.001**
On call for emergencies	2 (1–3)	124.88	2 (1–3)	122.82	**1 (1–2)**	**101.91**	**8.04**	**0.02**
Working on weekends	1 (1–2)	112.63	1 (1–2)	112.05	1 (1–2)	110.03	0.09	0.96
Forensics/cruelty investigations	3.5 (2.25–4)	135.91	3 (2–4)	116.94	**2 (1–4)**	**99.25**	**13.34**	**0.001**
Testifying in court	3 (2–4)	127.52	3 (1–4)	116.24	**2 (1–4)**	**102.82**	**6.30**	**0.04**
In house laboratory procedures	4 (3–4)	106.85	3 (3–4)	109.92	4 (3–4)	112.98	0.39	0.82
Development/fund raising	3 (2–3)	107.68	3 (2–4)	118.58	3 (1–4)	110.05	0.76	0.68
Humane education	4 (3–4)	99.88	4 (3–4)	104.41	4 (3–5)	117.42	3.64	0.16
Community education	4 (3–4)	97.59	4 (3–5)	107.77	4 (4–5)	117.33	4.18	0.12
Outreach clinics	4 (3.25–5)	107.64	5 (3–5)	125.24	4 (4–5)	108.07	2.67	0.26
Access-to-care clinics	4 (3–5)	109.09	4 (3–5)	121.67	4 (3–5)	108.57	1.46	0.48
Developing emergency preparedness plans	3.5 (3–4)	110.44	3 (3–4)	115.69	3.5 (2.75–4)	109.82	0.28	0.87
Staff training	4 (3–4)	124.53	4 (3–4)	117.41	4 (3–4)	103.67	4.89	0.09
Staff supervision	3 (2–4)	113.91	3 (2–4)	117.03	3 (2–4)	108.03	0.78	0.68

*Possible range from 1 (very unappealing) to 5 (very appealing)*.

*Bold text indicates there was a statistically significant difference based on Kruskal-Wallis H test and post-hoc pairwise comparisons with Bonferroni correction*.

Current and previous shelter veterinarians differed in the duties they performed as part of their job in shelter medicine. A significantly higher percentage of current shelter veterinarians undertook population management, euthanasia decision-making, administrative responsibilities, treatment decisions, adopt-ability decisions, health care and SOP development and forensics/cruelty investigations (Table 3).

**Table 3:** Frequency of common duties of shelter medicine among current and previous shelter veterinarians.

	**Shelter veterinarian (%)**	**Previous shelter veterinarian (%)**	***X*^**2**^**	***P* value**
Spay neuter	84.6	87.2	0.12	0.77
Pediatric spay/neuter	84.6	74.4	1.48	0.29
Other surgery	84.6	71.8	2.22	0.19
Population management	**69.2**	**46.2**	**4.92**	**0.03**
Euthanasia	78.8	64.1	2.43	0.16
Euthanasia decisions	**88.5**	**66.7**	**6.41**	**0.02**
Administrative responsibilities	**59.6**	**30.8**	**7.44**	**0.01**
Physical exams	90.4	87.2	0.23	0.74
Treatment decisions	**96.2**	**82.1**	**4.97**	**0.04**
Adopt-ability decisions	**71.2**	**46.2**	**5.83**	**0.02**
Behavior evaluation	40.4	30.8	0.89	0.39
Developing health care policies and SOPs	**78.8**	**56.4**	**5.27**	**0.04**
On-call for emergencies	50.0	35.9	1.80	0.21
Weekend hours	48.1	38.5	0.84	0.40
Forensics/cruelty investigations	**53.8**	**30.8**	**4.82**	**0.03**
Testifying in court	38.5	28.2	1.04	0.37
Laboratory procedures	38.5	25.6	0.53	0.61
Fund raising	19.2	25.6	0.53	0.61
Humane education	32.7	28.2	0.21	0.65
Community education	46.2	35.9	0.96	0.39
Outreach clinics	65.4	53.8	1.24	0.29
Access-to-care clinics	40.4	28.2	1.45	0.27
Emergency preparedness	32.7	20.5	1.66	0.24
Staff training	71.2	48.7	4.74	**0.05**
Staff supervision	67.3	51.3	2.39	0.14

*Bold text indicates there was a statistically significant difference based on Pearson Chi-Square (p < 0.05)*.

We also categorized veterinarians based on their desired work hours (fewer, the same, more) to examine the relationship between desired work hours and the appeal of being on-call or working weekend hours. In both cases, the relationship was not statistically significant (≥0.63).

### Characteristics of Shelter Medicine

Table 4 shows how characteristics of shelter medicine encouraged or discouraged current, previous, and non-shelter veterinarians' from working in the field. Previous shelter veterinarians had significantly lower mean rankings regarding the impact of promoting animal welfare, the ability to access employee benefits, opportunities for career development, and the availability of mentorship. They also had lower rankings for their interactions with administrative staff, their interactions with shelter veterinarians/staff, and the ability to be part of a multiple veterinarian team. Non-shelter veterinarians had lower mean ranks regarding the importance of their ability to promote animal welfare in shelter medicine, their interactions with shelter veterinarians/staff, their confidence in performing common shelter medicine procedures and the ability to perform duties without interacting with pet owners. In other words, current shelter veterinarians were encouraged to seek employment in shelter medicine based on these attributes at a higher rate than previous and non-shelter veterinarians.

**Table 4:** Kruskal-Wallis tests describing differences in the impact of characteristics of shelter medicine between current, previous, and non-shelter veterinarians.

	**Current shelter veterinarians**		**Previous shelter veterinarians**		**Non-shelter veterinarians**		***X*^**2**^**	***P***
	**Median**	**Mean rank**	**Median**	**Mean rank**	**Median**	**Mean rank**		
Salary expectations	2 (2–4)	107.02	3 (2–3)	112.10	3 (2–4)	112.26	0.28	0.87
Employee benefits	4 (3–5)	125.24	**3 (3–4)**	**71.97**	4 (3–5)	117.01	**19.96**	**<0.001**
Access to loan forgiveness	3 (3–5)	114.87	3 (3–4)	96.19	3 (3–5)	113.90	3.14	0.21
Regularity of work hours	4 (4–5)	122.43	4 (3–4)	92.19	4 (3–5)	112.07	0.57	0.06
Number of work hours	4 (3–4)	118.80	3 (3–4)	100.79	3 (3–4)	110.94	1.94	0.38
Workload	3 (2–4)	108.31	3 (2–4)	114.47	3 (2–4)	111.03	0.22	0.89
Promote animal welfare	5 (5–5)	142.05	**4 (4–5)**	**110.15**	**4 (4–5)**	**98.83**	**20.44**	**<0.001**
Provide community service	4 (4–5)	123.47	4 (4–5)	108.86	4 (4–5)	106.65	3.06	0.22
Opportunities for career development	4 (3–4.75)	127.27	**3 (2–4)**	**77.67**	4 (3–4)	114.49	**15.66**	**<0.001**
Perception of shelter medicine	3 (3–4)	116.35	3 (2–3)	101.92	3 (3–3)	111.58	1.44	0.49
Ability to find internship/ residency	3 (3–3.75)	119.87	3 (3–3)	102.85	3 (3–3)	109.90	2.59	0.27
Ability to find suitable jobs	4 (2–4)	118.59	3 (2–4)	110.85	3 (3–4)	108.01	1.11	0.58
Location of shelters	3 (3–4)	114.36	3 (3–4)	103.58	3 (3–4)	111.88	0.77	0.68
Opportunity to educate/ interact with owners	4 (3–4)	107.63	3 (3–4)	95.27	4 (3–4)	117.07	4.10	0.13
Opportunity to perform duties without interacting with pet owners	4 (4–5)	134.20	4 (3–5)	110.71	**4 (3–5)**	**101.81**	**10.46**	**0.01**
Strong emphasis on shelter live release rates	4 (3–4)	114.91	4 (3–4)	114.90	4 (3–4)	108.27	0.63	0.73
Risk of compassion fatigue, burnout or stress	2 (2–3)	114.02	2 (2–3)	125.62	2 (2–3)	105.41	3.66	0.16
Confidence in performing procedures	5 (4–5)	148.82	4 (4–5)	130.55	**4 (3–4)**	**90.01**	**39.73**	**<0.001**
Organizational policies/procedures	3 (2–4)	121.03	3 (2–3)	99.37	3 (2–4)	110.48	2.77	0.25
Interactions with administrative staff	3 (2–4)	116.53	**3 (2–3)**	**84.71**	3 (3–4)	116.68	**8.92**	**0.01**
Availability of mentorship	3 (3–4)	110.04	**3 (3–4)**	**87.91**	4 (3–4)	118.31	**7.55**	**0.02**
Ability to be part of a multiple veterinarian team	4 (4–5)	126.24	**3 (3–5)**	**89.10**	4 (3.75–5)	111.47	**8.48**	**0.01**
Interactions with shelter veterinarians/staff	4 (4–5)	137.33	**4 (3–5)**	**105.15**	**4 (3–4)**	**102.22**	**13.08**	**0.001**

*Possible range from 1 (strongly discourage) to 5 (strongly encourage). Bold text indicates there was a statistically significant difference based on Kruskal-Wallis H test and post-hoc pairwise comparisons with Bonferroni correction*.

We then grouped veterinarians based on their current outstanding loan debt and their loan debt at graduation (<$50,000, $50,000–$149,999 and ≥150,000). With increasing student loan debt (both current and at the time of graduation), we found the importance of employee benefits (*p* ≤ 0.03) and loan forgiveness increased (*p* < 0.001). There was no significant relationship between current or graduation loan debt and the importance of salary (*p* ≥ 0.11). Veterinarians' desired work hours (fewer, the same, more) were also not associated with the importance of the number and regularity of work hours when considering employment in shelter medicine (≥0.21).

### Job Satisfaction, Loneliness, and Professional Fulfillment

**Figure 1:**
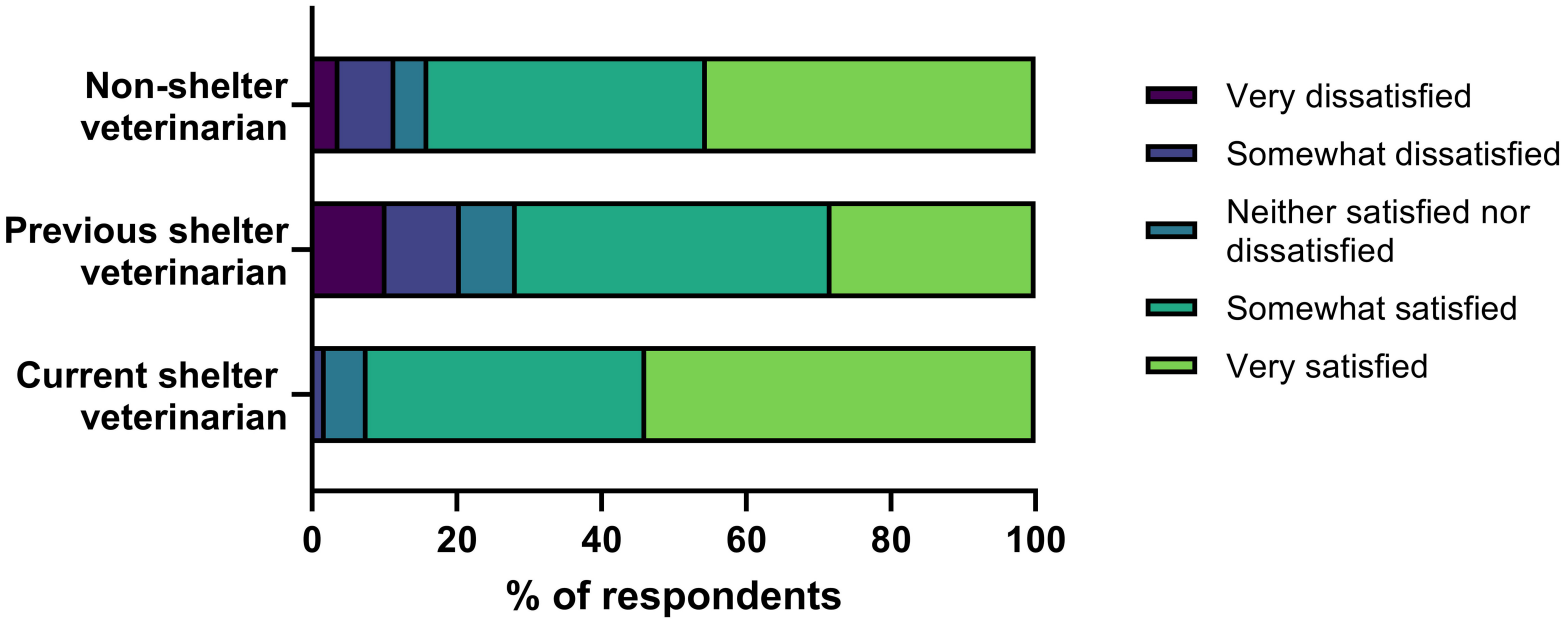
Job satisfaction in veterinary medicine.

There was a significant difference in job satisfaction between the 3 groups of veterinarians (*X*^2^ = 9.14, *p* = 0.01), with *post hoc* analyses indicating there was a significant difference between current and previous shelter veterinarians (Figure 1). Current shelter veterinarians had a median response of 5 (“very satisfied,” IQR 4-5) and mean rank of 125.74. Previous shelter veterinarians had a median response of 4 (“somewhat satisfied,” IQR 3-5) and a mean rank of 88.13. There was no significant difference between non-shelter veterinarians and either of the two groups of shelter veterinarians.

Current, previous, and non-shelter veterinarians reported relatively low levels of loneliness at work with a median score of 4 (possible range 3–9) and there was no statistically significant difference between the groups (*X*^2^ = 4.82, *p* = 0.09). Considering the loneliness questions individually, we found current, previous, and non-shelter veterinarians did not differ in their feelings of companionship (*X*^2^ = 3.20, *p* = 0.53) or isolation at work (*X*^2^ = 5.10, *p* = 0.28). Most veterinarians said they hardly ever lacked companionship at work (53%), although 37% said they lacked companionship some of the time and 10% lacked companionship often. Similarly, 60% of veterinarians hardly ever felt isolated from others, 30% felt isolated some of the time and 10% often felt isolated. However, there was a significant difference between the groups in terms of feeling left out at work (*X*^2^ = 12.43, *p* = 0.02), with a greater proportion of previous shelter veterinarians (21%) reporting they often felt left out compared with 15% of current shelter veterinarians and 5% of non-shelter veterinarians.

**Figure 2:**
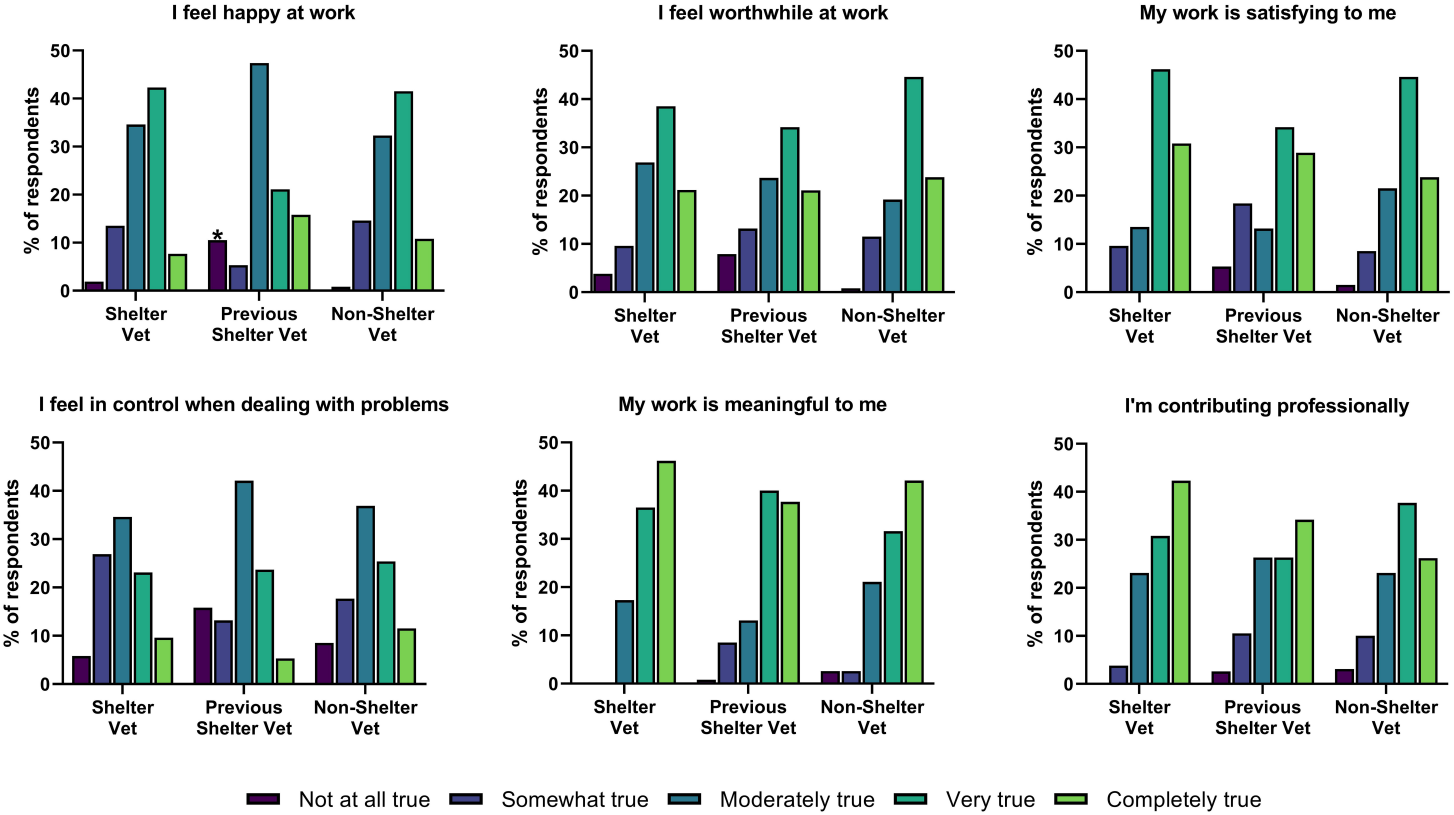
Professional fulfillment in veterinary medicine.

Veterinarians' feelings of professional fulfillment are shown in Figure 2. Current, previous, and non-shelter veterinarians differed in terms of how often they felt happy at work (*X*^2^ = 16.60, *p* = 0.02), with significantly more previous shelter veterinarians reporting they did not feel happy at work at all. There were no differences between the groups in the total professional fulfillment score [*F*_(2, 217)_ = 0.83, *p* = 0.44] with a mean response of 2.66 (possible range 0–4).

## Discussion

The goal of this study was to investigate the attributes of employment in shelter medicine that contribute to retention of veterinarians in the field. For the most part, there were no significant differences in the appeal of common shelter medicine duties between current and previous shelter veterinarians which suggests respondents were not motivated to leave shelter medicine due to a dislike of the job *per se*. The one exception was population management which was rated as significantly more appealing among current veterinarians compared with previous shelter veterinarians. A higher proportion of current shelter veterinarians in this study performed population management compared with previous shelter veterinarians, so it is possible that previous shelter veterinarians provided lower rankings due to their potentially limited understanding of what population management entails. It is also possible that previous shelter veterinarians were not exposed to population management if they left the field years ago, or that some veterinarians were required to undertake population management that would not be considered best practice by today's standards. The differing perceptions of population management between current and previous shelter veterinarians provides an interesting question for future research.

Non-shelter veterinarians found many of the common shelter medicine duties significantly less appealing than the other two groups, although 25% of non-shelter veterinarians indicated they were likely or extremely likely to seek employment in shelter medicine. More than half the previous shelter veterinarians also reported interest in shelter medicine which emphasizes the potential for animal shelters to attract these veterinarians to open shelter positions in the right conditions.

Despite the comparable ratings of shelter medicine duties between current and previous shelter veterinarians, we found significant differences in the duties they performed as part of their jobs. More current shelter veterinarians made euthanasia decisions, treatment decisions and adopt-ability decisions, performed administrative responsibilities, population management, forensics investigations, and staff training, and developed health care policies and/or SOPs, suggesting these duties may be important for job satisfaction and retention. In particular, it seems veterinarians who can participate in decision-making regarding individual patients' treatments and outcomes and shelter management policies may be more inclined to continue working in shelter medicine. Previous research in animal shelters has found staff involvement in euthanasia-decision making could help to reduce occupational stress related to euthanasia (12). Employee participation in decision-making has also been associated with job satisfaction, commitment, and effort across other industries (13, 14). The differing duties of current and previous shelter veterinarians could also be explained, at least in part, by the evolving nature of the field. Between 2011 and 2018, the frequency of veterinarians who made adopt-ability decisions, developed health care policies, and/or SOPs, testified in court, and participated in forensics/cruelty investigations increased significantly (3). It is therefore possible that veterinarians who left the field years ago did not have the opportunity to perform these duties.

Current shelter veterinarians, like previous and non-shelter veterinarians, reported being on call for emergencies and working on weekends were highly discouraging when they considered their employment in shelter medicine. These responsibilities appear to be universally unappealing, so minimizing these tasks could help to boost recruitment and retention of shelter veterinarians.

Our findings suggest that veterinary retention in shelter medicine is also impacted by employment characteristics, particularly those related to career development and workplace relations. Previous shelter veterinarians indicated their opportunities for career development and the availability of mentorship in shelter medicine deterred them from seeking employment in the field more so than current and non-shelter veterinarians. Employees' perceived career opportunities and the availability of career mentoring has been shown to predict employee turnover in non-animal related services (15, 16). Students and veterinarians have also recognized the importance of mentorship in the veterinary profession (17, 18). However, in a New Zealand study of recent veterinary graduates, almost half of the respondents reported they did not regularly meet with their supervisor to discuss their work or have a clear plan to develop their skills or experience. Not surprisingly, inadequate support was one of the key reasons that new graduates in the study had left their employment position (19). Many U.K. veterinary graduates have also described inadequate support from mentoring veterinarians (20). However, mentorship requires veterinarians to provide additional time and support and many veterinarians do not have sufficient training or resources to support new graduates (21). Taken together, these findings illustrate the importance of mentorship in veterinary practice and suggest animal shelters should endeavor to provide robust mentorship programs to increase recruitment and retention.

Previous shelter veterinarians also had lower ratings regarding their interactions with administrative staff and shelter veterinarians/veterinary staff, and their ability to be part of a multiple veterinarian team. A lack of peer support is a common workplace stressor for veterinarians, particularly female veterinarians (22). Interpersonal conflict in veterinary teams has been associated with the occurrence of workplace bullying, poor mental health, poor physical health and increased turnover intention (23). Toxic work environments have also been associated with decreased job satisfaction, increased cynicism, and burnout in veterinary medicine (9). Evidence also suggests workplace social support networks are crucial for veterinarians in the management of occupational stress and burnout (6, 9). Scotney, McLaughlin et al. (6) suggested the positive impact of a strong social support system at work may counteract the negative feelings of stress and burnout. Therefore, previous shelter veterinarians in the current study that felt ill-supported by administrative staff and their veterinary colleagues may have been more susceptible to burnout, stress, and compassion fatigue; phenomena that are all prevalent in the field of shelter medicine (6, 7, 24). Future research is needed to determine the prevalence and impact of workplace conflict in animal shelters and to develop interventions targeting improved workplace relations.

The importance of accessing employee benefits was also ranked significantly lower among previous shelter veterinarians compared with other veterinarians. It is not clear whether the ability to access employee benefits was simply less important for previous shelter veterinarians when considering their willingness to work in the field or whether previous shelter veterinarians believed the field of shelter medicine did not provide adequate employee benefits. The provision of employee benefits has increased significantly over recent years (3), so shelter veterinarians that left the field years ago may not have had access to the range of benefits that are offered to veterinarians today.

Veterinarians' ratings regarding the importance of accessing employee benefits and loan forgiveness programs were also impacted by their outstanding student loan debt and level of debt at the time of graduation. In both cases, veterinarians with a higher level of debt were more likely to indicate the provision of employee benefits and loan forgiveness programs encouraged them to seek employment in shelter medicine. Interestingly, the role of salary was not impacted by loan debt which suggests veterinarians with high loan debt placed an increased importance on loan forgiveness and benefits, perhaps due to the volume and burden of debt, compared with smaller differences in salary. Our findings support the increasing provision of employee benefits in the field (3) and the continued application of public service loan forgiveness programs. Animal shelters could also highlight the ability to access loan forgiveness programs as a benefit of the job when recruiting shelter veterinarians.

While the vast majority of veterinarians in this study were satisfied with their job, mirroring previous research from Australia (25), job satisfaction was significantly lower among previous shelter veterinarians. A higher proportion of previous shelter veterinarians also reported they did not feel happy at work and often felt left out which may be attributable, at least in part, to exclusion from the decision-making process and leadership roles. Although the overall scores for the loneliness scale were similar between the groups, 47% of veterinarians indicated they lacked companionship at least some of the time. Loneliness at work has been associated with emotional withdrawal from the employer and decreased work performance (26, 27). In veterinary medicine, poor job performance could have dire consequences for patients, including disability or death, which further emphasizes the need for animal shelters to address workplace culture and team relations.

Levels of professional fulfillment were not significantly different between the groups of veterinarians, although we found there were no current shelter veterinarians who felt their work was not at all satisfying or meaningful or who felt they were not contributing professionally compared with previous and non-shelter veterinarians. It seems current shelter veterinarians in this study recognized the importance of their work in shelter medicine which could benefit their well-being and job satisfaction. Future research is needed to further explore workplace satisfaction among shelter veterinarians using additional established questionnaires, such as the Gallup Employee Engagement survey. Veterinarians' feelings of professional fulfillment in this study were also comparable to previous reports from veterinary technicians (28) and human healthcare physicians (8).

Our data suggests the number of work hours was not a key factor driving turnover in shelter medicine. When asked if respondents would change the number of work hours per week, we found comparable responses between current, previous, and non-shelter veterinarians. Although, one quarter of veterinarians wished to work fewer hours for less total compensation. Data from the American Veterinary Medical Association (AVMA) has shown the percentage of veterinarians who want to work fewer hours per week increased between 2014 and 2018. Our findings suggest this trend is continuing. The AVMA reported 20% of veterinarians wished to reduce their weekly work hours in 2019 (29), compared with 25% in the current study in 2020–21. Like the AVMA, we found there was negative underemployment in veterinary medicine meaning there were more veterinarians who wished to work fewer hours per week than there were veterinarians who wished to work more hours per week. Expanding the veterinarian workforce is crucial to allow veterinarians to work at their optimal level and reduce burnout or stress (22, 30).

This study is the first of its kind to investigate retention of veterinarians in shelter medicine relative to common duties and characteristics of shelter medicine, and feelings of professional fulfillment and loneliness. The questionnaire was relatively comprehensive, and the study sample included veterinarians from various countries, universities, and fields of veterinary medicine. However, the study is also subject to some shortcomings. The breadth of questions in the survey meant we could not perform an in-depth analysis of the relationship between individual factors and retention. For instance, burnout is likely to play a role in turnover intention in the veterinary field, although we did not implement a validated tool to assess burnout. Nonetheless, the data provided in this study opens the door for future research to expand upon our findings. Recall bias may also have impacted our findings, particularly for previous veterinarians that left the field many years prior to the survey. We used various avenues to advertise the study and recruit veterinarians, although the sample size was limited which hinders the generalizability of the results. The study sample also included an overrepresentation of veterinarians from the University of Pennsylvania, likely due to the use of Penn Vet social media and newsletter postings to recruit participants. We also found the vast majority of respondents were Caucasian which mirrors the current racial profile of the veterinary workforce in the U.S. (31), but also reinforces the long-standing need for racial diversity in veterinary medicine (32). Cultural and language barriers in veterinary medicine deter individuals from accessing veterinary care, possibly due to a fear of being judged, exploited, or not being understood (33). Many shelters serve diverse populations, including minorities and underserved individuals, which further emphasizes the need for increased representation and recruitment of racially diverse veterinarians in shelter medicine.

## Conclusion

We investigated the perceptions of shelter medicine and feelings of job satisfaction, professional fulfillment, and loneliness among current, previous and non-shelter veterinarians. Our findings suggest that the appeal of shelter medicine duties was not a primary factor driving veterinarians to leave the field, although the ability to partake in duties related to shelter management and decision-making for individual patients appeared to be associated with retention of veterinarians in shelter medicine. Characteristics of employment related to career development and team conflict also appeared to impact the turnover of shelter veterinarians. Loneliness and professional fulfillment were comparable across the different fields of veterinary medicine, although previous shelter veterinarians were more likely to report they felt unhappy and left out at work. Animal shelters should employ strategies to improve workplace relationships and offer career development opportunities to improve job satisfaction and retention of veterinarians within the field.

## Data Availability Statement

The raw data supporting the conclusions of this article will be made available by the authors, without undue reservation.

## Ethics Statement

The studies involving human participants were reviewed and approved by the University of Pennsylvania Institutional Review Board. The patients/participants provided their written informed consent to participate in this study.

## Author Contributions

LP, CR, JS, and BW designed the study and interpreted the data. LP collected the data and conducted the statistical analyses and drafted the manuscript. All authors contributed to manuscript revision, and read and approved the submitted version.

## Conflict of Interest

The authors declare that the research was conducted in the absence of any commercial or financial relationships that could be construed as a potential conflict of interest.

## Publisher's Note

All claims expressed in this article are solely those of the authors and do not necessarily represent those of their affiliated organizations, or those of the publisher, the editors and the reviewers. Any product that may be evaluated in this article, or claim that may be made by its manufacturer, is not guaranteed or endorsed by the publisher.
