# Supplementary material for: Workplace Relations and Opportunities for Career Development Impact the Retention of Veterinarians in Shelter Medicine
Source: Front Vet Sci. 2021 Aug 25;8:732105. doi: 10.3389/fvets.2021.732105 (PMC8424191; doi:10.3389/fvets.2021.732105)
Supplement: Supplementary file 1 [file Data_Sheet_1.pdf]

Survey preamble: For the context of this survey, the terms “shelter” and “shelter medicine” refer to any job that is associated with the care of homeless animals or work in the community surrounding at-risk populations. This includes veterinarian roles in animal shelters, low-cost clinics and spay/neuter clinics.

1. What is your gender?
    - Male
    - Female
    - Other (open answer)

2. What is your age?
    - 20-29
    - 30-39
    - 40-49
    - 49 and older

3. What is your race?
    - American Indian/Alaskan Native
    - Native Hawaiian/Pacific Islander
    - Asian
    - Black or African American
    - Hispanic or Latino
    - White
    - Mixed race
    - Prefer not to answer
    - Unknown

4. What is your current salary before taxes?
    - <$50,000
    - $50,000-$99,999
    - $100,000-$149,999
    - $150,000-$199,999
    - ≥$200,000
    - Prefer not to answer

ⓘ *If you are contracted on an hourly basis, please provide an estimate of your yearly salary.*

5. a) What is your current outstanding student loan debt? (drop-down menu)
   b) What was your outstanding student loan debt when you graduated from veterinary school? (Drop down menu)
    - None
    - <$50,000
    - $50,000-$99,999
    - $100,000-$149,999
    - $150,000-$199,999
    - $200,000-$249,999

- $250,000-$299,999
- $300,000-$349,999
- $350,000-$399,999
- $400,000-$449,999
- $450,000-$499,999
- ≥$500,000
- Prefer not to answer

6. What year did you obtain your veterinary degree? (Open answer)

7. What school did you obtain your veterinary degree from? (Drop down menu)
    - Auburn University, Alabama
    - Tuskegee University, Alabama
    - Midwestern University, Arizona
    - Murdoch University, Australia
    - University of Melbourne, Australia
    - University of Queensland, Australia
    - University of Sydney, Australia
    - University of California, California
    - Western University of Health Sciences, California
    - University of Calgary, Canada
    - University of Guelph, Canada
    - Université de Montréal, Quebec, Canada
    - University of Prince Edward Island, Canada
    - University of Saskatchewan, Saskatchewan, Canada
    - Colorado State University, Colorado
    - VetAgro Sup, France
    - University of Florida, Florida
    - University of Georgia, Georgia
    - University of Illinois, Illinois
    - Purdue University, Indiana
    - Iowa State University, Iowa
    - University College Dublin, Ireland
    - Kansas State University, Kansas
    - Seoul National University, Korea
    - Louisiana State University, Louisiana
    - Tufts University, Massachusetts
    - Universidad Nacional Autonoma de México, Mexico
    - Michigan State University, Michigan
    - University of Minnesota, Minnesota
    - Mississippi State University, Mississippi
    - University of Missouri-Columbia, Missouri
    - Cornell University, New York
    - Long Island University, New York
    - State University of Utrecht, The Netherlands
    - Massey University College of Sciences, New Zealand

- North Carolina State University, North Carolina
- The Ohio State University, Ohio
- Oklahoma State University, Oklahoma
- Oregon State University, Oregon
- University of Pennsylvania, Pennsylvania
- University of Edinburgh, Scotland
- University of Glasgow, Scotland
- University of Tennessee, Tennessee
- Lincoln Memorial University, Tennessee
- Texas A&M University, Texas
- University of Bristol, United Kingdom
- University of London, United Kingdom
- Virginia Tech, Virginia
- Washington State University, Washington
- University of Wisconsin-Madison, Wisconsin
- Ross University, West Indies
- St George's University, West Indies
- Other (open answer - please specify)

8. Have you completed further education in addition to your veterinary degree? (Select all that apply)
    - Master's
    - Master of Public Health/Master of Preventive Veterinary Medicine
    - PhD
    - Board certification
    - Internship training program
    - Residency training program
    - Other
    - None

9. Are you currently completing, or do you intend to complete further education in addition to your veterinary degree? (Select all that apply)
    - Master's
    - Master of Public Health/Master of Preventive Veterinary Medicine
    - PhD
    - Board certification
    - Internship training program
    - Residency training program
    - Other
    - None

10. In what state or territory is your current primary employment? (Drop down menu including all states and territories and an option for 'Outside US' with open answer)

11. What field of veterinary medicine is your current primary employment?
    - Small animal

- Mixed practice
- Large animal
- Shelter medicine
- Equine
- Academia
- Government
- Exotics
- Laboratory animal
- Research
- Regulatory/Policy
- Zoo
- Other

ⓘ *Mixed practice includes at least 25% companion animal and at least 25% large animal or equine.*

12. On what basis are you currently employed at your primary employment setting?
    - Full time
    - Part-time (<35 hrs/week)
    - Casual
    - Volunteer
    - Other

Skip logic: if the participant indicated they currently work in “shelter medicine” in question 11, they skip to Stream A. If the participant indicated they do not currently work in shelter medicine in question 11, they are directed to question 13.

13. Have you ever been employed in the shelter medicine field?
    - Yes
    - No

Skip logic: if the participant answered ‘yes’ in question 13, they are directed to Stream B. If the participant answered ‘no’, they are directed to Stream C.

Stream A – Currently employed in shelter medicine

14. How long have you been employed as a veterinarian in the shelter medicine field?
    - Less than 1 year
    - 1-2 years
    - 3-5 years
    - 5-10 years
    - 11-20 years
    - More than 20 years

15. Overall, how satisfied are you with your current job in shelter medicine?

1<br>Very dissatisfied	2<br>Somewhat dissatisfied	3<br>Neither satisfied nor dissatisfied	4<br>Somewhat satisfied	5<br>Very satisfied

16. Please select all of the duties that you are required to perform as part of your job in shelter medicine. (Select all that apply)
    - Spay/neuter
    - Pediatric spay/neuter
    - Other surgery
    - Population management
    - Humane euthanasia
    - Euthanasia decision making process
    - Administrative responsibilities
    - Physical exams
    - Treatment decisions
    - Adopt-ability decisions
    - Behavior evaluation
    - Development of health care policies and/or standard operating procedures
    - On call/emergency duties
    - Weekend hours
    - Forensics/cruelty investigation
    - Testify in court
    - Performing laboratory procedures (fecal, blood smears, cytology, skin scrapes, etc.) in house
    - Development/fund raising
    - Humane education
    - Community education
    - Community outreach clinics (e.g., microchip, vaccination drives)
    - Access to care veterinary clinics
    - Emergency preparedness planning
    - Staff training
    - Staff supervision

17. How much do the following aspects of work in shelter medicine appeal to you?

	**1**<br>Very unappealing	**2**<br>Somewhat unappealing	**3**<br>Neither appealing nor unappealing	**4**<br>Somewhat appealing	**5**<br>Very appealing
Performing spay/neuter					
Performing pediatric spay/neuter					

Performing other surgery						
Managing populations						
Performing humane euthanasia						
Making euthanasia decisions						
Performing administrative responsibilities						
Performing physical exams						
Making treatment decisions						
Making adopt-ability decisions						
Evaluating behavior						
Developing health care policies and/or standard operating procedures						
Being on call for emergencies						
Working on weekends						
Performing forensics/cruelty investigations						
Testifying in court						
Performing laboratory procedures (fecal, blood smears, cytology, skin scrapes, etc.) in house						
Participating in development/fund raising						
Providing humane education						
Providing community education						

Participating in community outreach clinics (e.g., microchip, vaccination drives)					
Participating in access to care community clinics					
Developing emergency preparedness plans					
Training staff					
Supervising staff					

18. How much do the following factors discourage or encourage you to continue working in the shelter medicine field?

N.B. Below options will be presented in a randomized order in Qualtrics.

	**1** Strongly discourage	**2** Somewhat discourage	**3** No influence	**4** Somewhat encourage	**5** Strongly encourage
Salary expectations					
Ability to access employee benefits, e.g., health insurance					
Ability to access loan forgiveness program					
Regularity of work hours					
Number of work hours					
Workload					
Ability to promote animal welfare					
Ability to provide community service					
Opportunities for career development					
The perception of shelter medicine among other veterinary disciplines					
Ability to find a suitable internship or residency					
Ability to find suitable shelter veterinarian jobs					

Location of shelters					
Opportunity to educate and interact with pet owners					
Ability to perform duties without interacting with pet owners					
Strong emphasis on shelter live release rates					
Risk of compassion fatigue, burnout or occupational stress					
Confidence in performing shelter medicine procedures					
Organizational policies and procedures					
Interactions with administrative staff					
Availability of mentorship					
Ability to be part of a multiple veterinarian team					
Interactions with shelter veterinarians or veterinary staff					

19. Are there any additional reasons not described above that you chose to work in shelter medicine?
    - Yes
    - No

If answered yes, please describe the additional reasons that you chose to work in shelter medicine. (Open answer)

20. Are there any additional reasons not described above that you have considered leaving shelter medicine?
    - Yes
    - No

If answered yes, please describe the additional reasons that you have considered leaving shelter medicine. (Open answer)

21. If you could change the number of hours you work per week, would you:
    - Work more hours per week for a higher level of total compensation
    - Work fewer hours per week for a lower level of total compensation

- Work the same number of hours per week with no change to your current compensation.

22. Answer the following questions regarding the social aspects of your professional life.

	**1**<br>Hardly ever	**2**<br>Some of the time	**3**<br>Often
At work, how often do you feel that you lack companionship?			
At work, how often do you feel left out?			
At work, how often do you feel isolated from others?			

23. Consider the following statements regarding your professional fulfilment.

	**1**<br>Not at all true	**2**<br>Somewhat true	**3**<br>Moderately true	**4**<br>Very true	**5**<br>Completely true
I feel happy at work					
I feel worthwhile at work					
My work is satisfying to me					
I feel in control when dealing with difficult problems at work					
My work is meaningful to me					
I’m contributing professionally in the ways I value most					

Stream B – Previously employed in shelter medicine

24. In what capacity did you work as a veterinarian in shelter medicine?
    - Full time
    - Part-time (<35 hrs/week)
    - Casual
    - Volunteer
    - Other

25. How long were you employed as a veterinarian in the shelter medicine field?
    - Less than 1 year
    - 1-2 years
    - 3-5 years
    - 5-10 years
    - 11-20 years
    - More than 20 years

26. When did you leave the shelter medicine field?
    - Less than 1 year ago
    - 1-2 years ago
    - 3-5 years ago
    - 5-10 years ago
    - 11-20 years ago
    - More than 20 years ago

27. How satisfied were you with your employment in the shelter medicine field?

1	2	3	4	5
Very dissatisfied	Somewhat dissatisfied	Neither satisfied nor dissatisfied	Somewhat satisfied	Very satisfied

28. How likely are you to consider working in shelter medicine in the future?

1	2	3	4	5
Extremely unlikely	Somewhat unlikely	Neither likely nor unlikely	Somewhat likely	Extremely likely

29. Please select all of the duties that you were required to perform as part of your job in shelter medicine. (Select all that apply)
    - Spay/neuter
    - Pediatric spay/neuter
    - Other surgery
    - Population management
    - Humane euthanasia
    - Euthanasia decision making process
    - Administrative responsibilities

- Physical exams
- Treatment decisions
- Adopt-ability decisions
- Behavior evaluation
- Development of health care policies and/or standard operating procedures
- On call/emergency duties
- Weekend hours
- Forensics/cruelty investigation
- Testify in court
- Performing laboratory procedures (fecal, blood smears, cytology, skin scrapes, etc.) in house
- Development/fund raising
- Humane education
- Community education
- Community outreach clinics (e.g., microchip, vaccination drives)
- Access to care veterinary clinics
- Emergency preparedness planning
- Staff training
- Staff supervision

30. How much did the following aspects of work in shelter medicine appeal to you?

	**1** Very unappealing	**2** Somewhat unappealing	**3** Neither appealing nor unappealing	**4** Somewhat appealing	**5** Very appealing
Performing spay/neuter					
Performing pediatric spay/neuter					
Performing other surgery					
Managing populations					
Performing humane euthanasia					
Making euthanasia decisions					
Performing administrative responsibilities					
Performing physical exams					
Making treatment decisions					

Making adopt-ability decisions					
Evaluating behavior					
Developing health care policies and/or standard operating procedures					
Being on call for emergencies					
Working on weekends					
Performing forensics/cruelty investigations					
Testifying in court					
Performing laboratory procedures (fecal, blood smears, cytology, skin scrapes, etc.) in house					
Participating in development/fund raising					
Providing humane education					
Providing community education					
Participating in community outreach clinics (e.g., microchip, vaccination drives)					
Participating in access to care community clinics					
Developing emergency preparedness plans					
Training staff					
Supervising staff					

31. How much did the following factors encourage or discourage you from working in the shelter medicine field?

N.B. Below options will be presented in a randomized order in Qualtrics.

	1<br>Strongly discourage	2<br>Somewhat discourage	3<br>No influence	4<br>Somewhat encourage	5<br>Strongly encourage
Salary expectations					
Ability to access employee benefits, e.g., health insurance					
Ability to access loan forgiveness program					
Regularity of work hours					
Number of work hours					
Workload					
Ability to promote animal welfare					
Ability to provide community service					
Opportunities for career development					
The perception of shelter medicine among other veterinary disciplines					
Ability to find a suitable internship or residency					
Ability to find suitable shelter veterinarian jobs					
Location of shelters					
Opportunity to educate and interact with pet owners					
Ability to perform duties without interacting with pet owners					
Strong emphasis on shelter live release rates					
Risk of compassion fatigue, burnout or occupational stress					
Confidence in performing shelter medicine procedures					
Organizational policies and procedures					

Interactions with administrative staff					
Availability of mentorship					
Ability to be part of a multiple veterinarian team					
Interactions with shelter veterinarians or veterinary staff					

32. Are there any additional reasons not described above that you chose to work in shelter medicine?
    - Yes
    - No

If answered yes, please describe the additional reasons that you chose to work in shelter medicine. (Open answer)

33. Are there any additional reasons not described above that you chose to leave shelter medicine?
    - Yes
    - No

If answered yes, please describe the additional reasons that you chose to leave shelter medicine. (Open answer)

34. Considering your previous job in shelter medicine, if you could have changed the number of hours you worked per week, would you have:
    - Worked more hours per week for a higher level of total compensation
    - Worked fewer hours per week for a lower level of total compensation
    - Worked the same number of hours per week with no change to your compensation.

35. Answer the following questions regarding the social aspects of your previous job in shelter medicine.

	**1** Hardly ever	**2** Some of the time	**3** Often
At work, how often did you feel that you lacked companionship?			
At work, how often did you feel left out?			
At work, how often did you feel isolated from others?			

36. Consider the following statements regarding your professional fulfilment in your previous job in shelter medicine.

	**1**<br>Not at all true	**2**<br>Somewhat true	**3**<br>Moderately true	**4**<br>Very true	**5**<br>Completely true
I felt happy at work					
I felt worthwhile at work					
My work was satisfying to me					
I felt in control when dealing with difficult problems at work					
My work was meaningful to me					
I was contributing professionally in the ways I valued most					

Stream C – Never employed in shelter medicine

37. How satisfied are you with your current employment?

1	2	3	4	5
Very dissatisfied	Somewhat dissatisfied	Neither satisfied nor dissatisfied	Somewhat satisfied	Very satisfied

38. How likely are you to consider working in shelter medicine in the future?

1	2	3	4	5
Extremely unlikely	Somewhat unlikely	Neither likely nor unlikely	Somewhat likely	Extremely likely

39. How much do the following aspects of work in shelter medicine appeal to you?

	**1** Very unappealing	**2** Somewhat unappealing	**3** Neither appealing nor unappealing	**4** Somewhat appealing	**5** Very appealing
Performing spay/neuter					
Performing pediatric spay/neuter					
Performing other surgery					
Managing populations					
Performing humane euthanasia					
Making euthanasia decisions					
Performing administrative responsibilities					
Performing physical exams					
Making treatment decisions					
Making adopt-ability decisions					
Evaluating behavior					

Developing health care policies and/or standard operating procedures					
Being on call for emergencies					
Working on weekends					
Performing forensics/cruelty investigations					
Testifying in court					
Performing laboratory procedures (fecal, blood smears, cytology, skin scrapes, etc.) in house					
Participating in development/fund raising					
Providing humane education					
Providing community education					
Participating in community outreach clinics (e.g., microchip, vaccination drives)					
Participating in access to care community clinics					
Developing emergency preparedness plans					
Training staff					
Supervising staff					

40. How much would the following factors encourage or discourage you from working in the shelter medicine field?

N.B. Below options will be presented in a randomized order in Qualtrics.

	**1** Strongly discourage	**2** Somewhat discourage	**3** No influence	**4** Somewhat encourage	**5** Strongly encourage
Salary expectations					

Ability to access employee benefits, e.g., health insurance					
Ability to access loan forgiveness program					
Regularity of work hours					
Number of work hours					
Workload					
Ability to promote animal welfare					
Ability to provide community service					
Opportunities for career development					
The perception of shelter medicine among other veterinary disciplines					
Ability to find a suitable internship or residency					
Ability to find suitable shelter veterinarian jobs					
Location of shelters					
Opportunity to educate and interact with pet owners					
Ability to perform duties without interacting with pet owners					
Strong emphasis on shelter live release rates					
Risk of compassion fatigue, burnout or occupational stress					
Confidence in performing shelter medicine procedures					
Organizational policies and procedures					
Interactions with administrative staff					
Availability of mentorship					

Ability to be part of a multiple veterinarian team					
Interactions with shelter veterinarians or veterinary staff					

41. Are there any additional reasons not described above that would motivate you to work in shelter medicine?
    - Yes
    - No

If answered yes, please describe the additional reasons that would motivate you to work in shelter medicine. (Open answer)

42. Are there any additional reasons not described above that you chose not to work in shelter medicine?
    - Yes
    - No

If answered yes, please describe the additional reasons that you chose not to work in shelter medicine. (Open answer)

43. If you could change the number of hours you work per week in your current job, would you:
    - Work more hours per week for a higher level of total compensation
    - Work fewer hours per week for a lower level of total compensation
    - Work the same number of hours per week with no change to your current compensation.

44. Answer the following questions regarding the social aspects of your professional life.

	**1** Hardly ever	**2** Some of the time	**3** Often
At work, how often do you feel that you lack companionship?			
At work, how often do you feel left out?			
At work, how often do you feel isolated from others?			

45. Consider the following statements regarding your professional fulfilment.

	**1** Not at all true	**2** Somewhat true	**3** Moderately true	**4** Very true	**5** Completely true
I feel happy at work					
I feel worthwhile at work					

My work is satisfying to me<br><br>I feel in control when dealing with difficult problems at work<br><br>My work is meaningful to me<br><br>I'm contributing professionally in the ways I value most					
